# Oregon

**Published:** 1897-02

**Authors:** 


*Oregon.*—Three herds of Jersey cattle in this State have been found tuberculous, and a number were destroyed.

